# Intérêt des prothèses métalliques expansives dans la prise en charge de l’occlusion tumorale colique: expérience d’un service hospitalier Marocain

**DOI:** 10.11604/pamj.2013.14.68.1982

**Published:** 2013-02-18

**Authors:** Fedoua Rouibaa, Meriem Bakkar, Hassan Seddik, Tarik Addioui, Fatima Zahra Filali, Rachid Akka, Hanane Desla, Aourarh Aourarh

**Affiliations:** 1 Service de gastroenterologie 1, Hôpital Militaire d’Instruction Mohamed V, Rabat, Maroc

**Keywords:** Cancer colorectal, occlusion colique, prothèse métallique expansive, colorectal cancer, colonic obstruction, expanding metallic stent

## Abstract

Le traitement de l’obstruction colique d’origine tumorale s’est modifié ces dernières années grâce à l’apport des endoprothèses coliques. Elles constituent une alternative thérapeutique reconnue au traitement chirurgical dans 2 indications: la levée en urgence de l’occlusion colique aigue avant une chirurgie curatrice ou pour le traitement palliatif de l’obstruction colique chez les patients inopérables ou ayant une maladie localement avancée ou métastatique. Il s’agit de 12 observations de syndrome occlusif colique d’origine tumorale traitées par endoprothèses coliques au sein du service de Gastro-entérologie 1 de l’hôpital militaire d’instruction Mohammed V. L’âge moyen était de 53 ans. Il existait une prédominance masculine (9 hommes - 3 femmes). Tous les patients présentaient un syndrome occlusif avec arrêt des matières et des gaz. L’abdomen sans préparation montrait des niveaux hydro-aériques chez tous les patients. La tomodensitométrie objectivait un processus colique sténosant avec dilatation des anses en amont. Tous les patients ont bénéficié de la mise en place d’une endoprothèse par colonoscopie sous anesthésie générale. Le succès technique immédiat et clinique était noté dans 95% des cas. La migration de la prothèse a été notée dans un cas. Les endoprothèses coliques constituent une alternative de choix au traitement chirurgical des sténoses tumorales coliques symptomatiques, soit pour permettre de préparer au traitement curatif chirurgical “à froid”, soit dans un but palliatif avant de débuter une éventuelle chimiothérapie.

## Introduction

Le cancer du côlon est révélé par une occlusion digestive dans 8 à 29% des cas [1]. Le traitement des occlusions tumorales coliques était habituellement chirurgical. Il était grevé d’une morbidité et d’une mortalité élevée liées au caractère urgent de l’intervention chez des malades souvent âgés, en mauvais état général, avec un côlon non préparé [2]. L’utilisation des prothèses métalliques expansives (PME) pour lever les obstructions coliques malignes est une alternative qui a été proposée par DOHMOTO en 1991 [3]. Depuis, plusieurs auteurs ont rapporté des taux de succès sur la levée de l’obstacle atteignant 90%, avec une morbidité et une mortalité inférieures à celles du traitement chirurgical [4, 5]. La mise en place d’une endoprothèse métallique colique est utilisée comme alternative à la chirurgie en urgence dans deux situations: 1) le traitement palliatif définitif de l’occlusion en cas de contre-indication à une chirurgie curative liée à la présence de métastases non-résécables ou à un terrain très altéré; 2) le traitement provisoire d’urgence de l’occlusion permettant ainsi la résection chirurgicale de la tumeur en un temps sans colostomie lors de la même hospitalisation.

Le but de notre travail était de prouver la faisabilité, la sûreté et l’efficacité de l’utilisation des PME dans la prise en charge du cancer colorectal occlusif par une étude rétrospective de 12 prothèses consécutives.

## Méthodes

Entre juillet 2009 et avril 2012, douze malades ont été hospitalisés au sein du service de Gastro-entérologie I de l’Hôpital Militaire d’Instruction Mohammed V (HMIMV) pour une occlusion colique d’origine tumorale, et ont bénéficié de la mise en place d’endoprothèse colique métallique.

**Figure 1:**
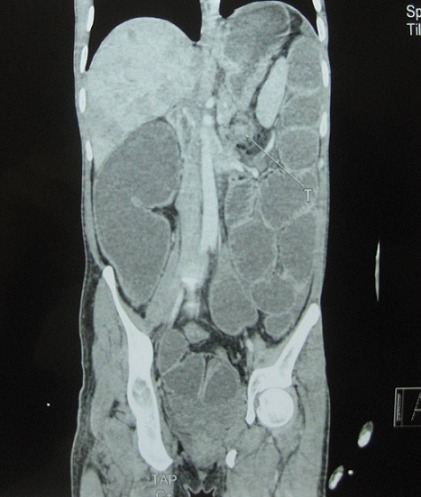
TDM abdominale: tumeur colique avec dilatation d’amont

Tous les malades ont bénéficié d’une évaluation clinique avec mise en condition et correction des troubles électrolytiques et de la dénutrition qui ont été fréquemment associés; Le bilan radiologique a reposé sur les clichés de l’abdomen sans préparation et sur la TDM abdominale (Figure 1). Les variables étudiées: âge, sexe, indication (palliatif ou bridge), stade TNM, traitement complémentaire.

**Figure 2:**
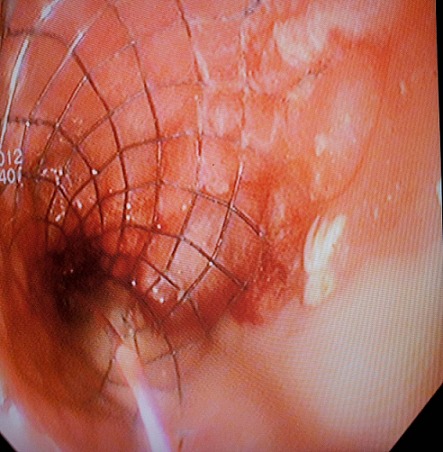
Vue endoscopique d’une prothèse colique après largage

**Figure 3:**
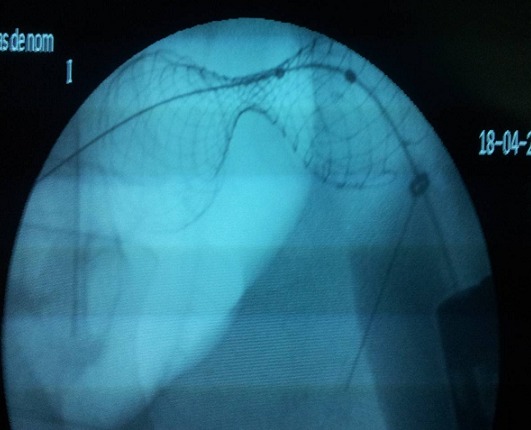
Endoprothèse colique en place sous contrôle radiologique après largage

La colonoscopie a été réalisée sous anesthésie générale, après préparation colique par voie basse, le malade étant en décubitus dorsal. L’endoscope est poussé très prudemment jusqu’au pôle inférieur de la sténose tumorale (dont la distance par rapport à la marge anale a été évaluée) avec une insufflation minimale. L’opacification par du produit de contraste hydrosoluble à l’aide d’un cathéter monté sur fil guide hydrophile permet de visualiser la sténose, d’estimer précisément la longueur tumorale et de déterminer la longueur de l’endoprothèse à utiliser, puis la prothèse est larguée sous contrôle endoscopique et radioscopique(Figure 2, Figure 3).

Une radiographie de l’abdomen sans préparation était réalisée chez tous les malades après largage de la prothèse afin de vérifier son emplacement et surtout pour éliminer une complication à type de perforation colique au moment du geste.

## Résultats

Il s’agit d’une série de douze malades (9 hommes et 3 femmes) hospitalisés au service de Gastro-entérologie I de l’hôpital Militaire d’Instruction Mohamed V pour occlusion colique d’origine tumorale maligne, qui ont bénéficié de la pose d’endoprothèse métallique expansive.

L’âge moyen des patients était de 53 ans (49 -71ans). Le diagnostic a été fait sur les données cliniques: douleurs abdominales, arrêt des matières et gaz, distension abdominale, ampoule rectale vide; Et radiologiques: la radiographie d’abdomen sans préparation qui a montré des niveaux hydro-aériques de type colique chez tous les patients, et la TDM abdominale qui a mis en évidence le processus colique sténosant avec dilatation des anses en amont. La sténose siégeait au niveau de la charnière recto-sigmoïdienne chez 5 malades, dans le colon gauche chez 4 malades et au niveau du haut rectum chez 3 malades. L’indication du traitement par endoprothèse était réalisée en intention palliative dans 10 cas (83%) et dans 2 cas en intention préopératoire.

Le taux de succès technique défini par la mise en place et le déploiement de la PMC était de 95%. Le succès clinique défini par la levée de l’occlusion colique dans les 48 heures, sans la nécessité d’une re-intervention a été noté dans 90%. Les suites immédiates ont été favorables chez tous les malades. Aucune complication immédiate n’a été mentionnée, et la durée moyenne du séjour hospitalier était de trois jours. Un cas de migration de prothèse est survenu chez un seul patient après un délai de 8 mois.

Le traitement réalisé dans les suites de la pose de prothèse colique était une chimiothérapie dans 44% des cas, aucun traitement n’était réalisé dans 36% des cas, une résection chirurgicale curative était pratiquée dans 2 cas, une stomie était de mise en place dans un cas.

## Discussion

Longtemps chirurgical, le traitement de l′obstruction colique d′origine tumorale s′est modifié ces dernières années grâce à l′apport des prothèses métalliques expansives (PME). Les PME constituent une alternative thérapeutique reconnue au traitement chirurgical dans 2 indications: la levée en urgence de l′occlusion colique aiguë avant une chirurgie curatrice (si elle est possible) en un temps dans de meilleures conditions environ 10 jours après [4–6]; le traitement palliatif de l′obstruction colique chez les patients ayant une maladie localement avancée ou métastasée, chez ceux dont l′état général est trop altéré pour une intervention chirurgicale [6].

L’indication de la mise en place d’une PME n’est actuellement bien validée que pour une tumeur occlusive située entre le haut rectum et le transverse gauche [6]. Le diagnostic de localisation de l’occlusion est porté sur la tomodensitométrie. Le toucher rectal permet, en cas de perception au doigt de la lésion, de contre-indiquer cette technique. La sténose tumorale est située dans plus de 60% des cas au niveau de la charnière rectosigmoidienne ou du sigmoïde comme c’est le cas dans notre série.

Le délai de réalisation de l’intervention est idéalement de moins de 12 heures. Le patient est installé sur une table de radiologie ou au bloc opératoire avec une scopie mobile. La vidange du colon distal permet dans la majorité des cas de progresser jusqu’à la sténose tumorale sans préparation colique. L’endoscope est poussé très prudemment jusqu’au pôle inférieur de la sténose tumorale avec une insufflation minimale. La nature tumorale maligne de la sténose est confirmée avec une grande probabilité dans la majorité des cas. L’opacification par du produit de contraste hydrosoluble permet de visualiser la sténose. Le trajet tumoral est cathétérisé avec un fil-guide hydrophile. Après franchissement par le cathéter, l’opacification d’amont permet d’estimer précisément la longueur tumorale et de déterminer la longueur de la PME à utiliser. Le cathéter doit être positionné suffisamment en amont de la sténose avant de le remplacer par un fil-guide rigide de 0,035 pouce [6]. Les PME actuellement les plus utilisées sont en nitinol et non couvertes. Dans notre expérience, les sténoses ont une longueur moyenne de 40 mm. Dans plus de 70% des cas, on était amené à utiliser des PME de 90 mm de longueur totale.

Le contrôle radioscopique et endoscopique du positionnement de la PME avant largage et la surveillance du largage sont fondamentaux. La PME doit être centrée par rapport à la sténose. Une débâcle immédiate de selles liquides suit l’expansion complète de la prothèse.

Différentes études comparatives (Prothèse colique définitive versus chirurgie) montraient un taux de succès technique de pose des PME et clinique de 95 et 90% [7] avec une durée d’hospitalisation inférieure, une médiane de survie identique et une médiane de perméabilité de 10 mois. Le suivi à long terme dans une étude prospective multicentrique de Faragher et al a montré que 86% des patients avaient une prothèse fonctionnelle sans complication jusqu′à leur décès [8].

Les complications tardives sont fréquentes: perforation (5-9%), Migration (5-25%), obstruction (10-15%). Les facteurs de risques de ces complications sont le plus souvent liés à l′étroitesse de la sténose, la localisation au niveau de l’angle colique gauche et du colon droit, la pneumatose colique préalable et le Bevacizumab pour la perforation [9].

Concernant l’efficacité de l’utilisation de la prothèse colique en pré-opératoire, une méta-analyse de Tilney et al [10] regroupant dix études comparatives (Chirurgie versus prothèse puis chirurgie) a montré une diminution significative de la durée de séjour et de la morbidité pour les patients traités par prothèse [11]. Cette étude concluait à la supériorité de la prothèse sur la chirurgie décompressive d’urgence en termes de morbidité liée à la procédure (6 vs 31%) et en termes de durée d’hospitalisation.

## Conclusion

La mise en place endoscopique des prothèses métalliques expansives devient une technique de référence dans la prise en charge de l’occlusion tumorale colique, avec un succès technique et clinique dans 90% des cas, que ce soit en préalable à une chirurgie réglée ou comme traitement palliatif. La nécessité de disposer d’une structure adaptée et d’une équipe d’endoscopie formée à cette technique a limité jusqu’ici sa mise en ‘uvre. La réunion de ces deux conditions permettra sa diffusion, constituant ainsi une nouvelle et séduisante attitude thérapeutique alternative à la chirurgie.
